# Gene‐centric analysis implicates nuclear encoded mitochondrial protein gene variants in migraine susceptibility

**DOI:** 10.1002/mgg3.270

**Published:** 2017-01-17

**Authors:** Shani Stuart, Miles C. Benton, David A. Eccles, Heidi G. Sutherland, Larisa M. Haupt, Rodney A. Lea, Lyn R. Griffiths

**Affiliations:** ^1^ Genomics Research CentreInstitute for Biomedical Health and InnovationSchool of Biomedical SciencesQueensland University of TechnologyBrisbaneQueensland4059Australia

**Keywords:** Complex disease, gene centric, migraine, mitochondrial dysfunction, neurogenetics, Norfolk Island, nuclear encoded mitochondrial protein genes

## Abstract

**Background:**

Migraine is a common neurological disorder which affects a large proportion of the population. The Norfolk Island population is a genetically isolated population and is an ideal discovery cohort for genetic variants involved in complex disease susceptibility given the reduced genetic and environmental heterogeneity. Given that the majority of proteins responsible for mitochondrial function are nuclear encoded, this study aimed to investigate the role of Nuclear Encoded Mitochondrial Protein (NEMP) genes in relation to migraine susceptibility.

**Methods:**

A gene‐centric association analysis of NEMP genes was undertaken in the most related individuals (*n* = 315) within the genetically isolated Norfolk Island population. The discovery phase included genes with three or more SNP associations (*P* < 0.005), which were investigated further in a replication phase using an unrelated migraine case–control cohort (544 patients and 584 controls).

**Results:**

The discovery phase of the study implicated SNPs in 5 NEMP genes to be associated with migraine susceptibility (*P* < 0.005). Replication analysis validated some of these implicated genes with SNPs in three NEMP genes shown to be associated with migraine in the replication cohort. These were *CSNK1G3* (*P* = 0.00037)*, ELOVL6* (*P* = 0.00035) and *SARDH* (*P* = 0.00081), which are involved in phosphorylation, fatty acid metabolism, and oxidative demethylation, respectively.

**Conclusion:**

Here we provide evidence that variation in NEMP genes is associated with migraine susceptibility. This study provides evidence for a link between mitochondrial function and migraine susceptibility.

## Introduction

The mitochondria is the energy producing organelle present in all mammalian cells, occurring in numbers proportional to the energy requirements of each specific tissue. Muscle fibers and neuronal networks have the highest energy requirements in the human body and each cell can contain several thousand mitochondria ensuring that the demand for ATP is met. Consequently genetic aberrations which inhibit the Oxidative Phosphorylation (OXPHOS) pathway have a profound impact on energy production and most adversely affect the muscle and neuronal cells (Sparaco et al. 2006). Deleterious mutations with high rates of penetrance have devastating effects and cause a range of myopathies and encephalopathies including: Mitochondrial Encephalomyopathy; lactic acidosis and stroke like episodes (MELAS) (Prasad et al. 2014), and Myoclonic Epilepsy with ragged red fibers (MERRF) (Wallace et al. 1988). Symptoms of these diseases include severe migraine attacks which has led researchers to hypothesize that mitochondrial dysfunction may be linked to the more common subtypes of migraine (Stuart and Griffiths 2012). Migraine is a costly disorder, being listed as one of the top twenty most debilitating diseases by the World Health Organisation (WHO), and presents significant economic and personal burden (Leonardi et al. 2005). Migraine affects approximately 12% of the adult general population and is classified by the International Headache Society into two main subtypes namely migraine without aura (MO) and migraine with aura (MA) (Olesen and Lipton 1994).

The mitochondria itself contains its own genome, made up of just 37 genes. These genes are transcribed to produce 22 tRNAs, two rRNAs and 13 polypeptides. The tRNA and rRNA molecules are involved in forming ribosomal units and translating the 13 polypeptides into proteins which form critical components of the OXPHOS subunits. Complex I is composed of 46 polypeptides of which seven are mitochondrially encoded, complex III is made up of just one of 11 mitochondrially encoded polypeptides and complex IV has three of 13. Similarly only two of 16 proteins in complex V are encoded by the mitochondria (Wallace 2005). All the other components of OXPHOS including the entire subunit II are encoded by nuclear genes. In addition to structural components of OXPHOS, all the mitochondrial metabolic enzymes, transcription factors, and other regulatory molecules which govern mitochondrial function are nuclear encoded (Pagliarini et al. 2008). Current estimates are that more than 1000 proteins are encoded by the nuclear DNA and imported into the mitochondrial matrix (Gioio et al. 2001). The vast majority of active molecules involved in mitochondrial function are imported into the matrix from the nucleus in this way with <1% of mitochondrial function attributed to the mitochondrial genome itself (Hendrickson et al. 2010). Through this understanding of mitochondrial function, it is clear that investigation of nuclear encoded mitochondrial protein (NEMP) genes is critical for discovering the underlying genetic cause of mitochondrial disorders.

The purpose of this study was to investigate the role of NEMPs in relation to migraine susceptibility. We hypothesized that mitochondrial dysfunction, which is influenced heavily by NEMP genes (Lu and Claypool 2015), could lower the threshold for a migraine attack and that by understanding the genetic etiology of this disease new approaches to migraine treatment/management may be identified. The link between mitochondrial dysfunction and migraine was first suggested in the 1980s and since then a growing body of evidence has strengthened this hypothesis (Sparaco et al. 2006; Stuart and Griffiths 2012; Yorns and Hardison 2013). Strong evidence from biochemical, morphological, and therapeutic studies show a link between mitochondrial dysfunction and migraine susceptibility (Montagna et al. 1994; Sangiorgi et al. 1994; Okada et al. 1998; Finnila et al. 2001; D'Andrea et al. 2006; Brenner 2010; Yorns and Hardison 2013). However, genetic studies have been limited by sample size and molecular data making this an area that needs to be addressed. This study aimed to use the genetically isolated Norfolk Island population as a discovery cohort for identification of NEMP genes involved with migraine susceptibility and to replicate significant findings in a large Australian Caucasian migraine patient‐control population to validate the role of identified variants in this common neurological disorder. Norfolk Island is a genetically isolated population situated off the coast of Australia, best known from the “Bounty on the Mutiny” historical account. This isolate is an ideal population for identification of complex disease traits as the genetic heterogeneity typical of these diseases is reduced. Geographical isolation further reduces environmental heterogeneity and environment‐gene interactions, increasing the chance of identifying true causal variants (Bellis et al. 2005; Macgregor et al. 2010).

## Materials and Methods

### Discovery phase – using the genetically isolated Norfolk Island population

#### Sample selection

The Norfolk Island population is a genetically isolated population situated off the east coast of Australia and is an ideal discovery cohort for genetic variants involved in complex disease susceptibility given the reduced genetic and environmental heterogeneity. The most related Core Pedigree individuals were selected to maximize the potential for discovering disease causing variation and *n* = 315 individuals were included in this study. The selected individuals consisted of 80 migraine sufferers and 235 healthy controls with migraine affected individuals occurring in large family subbranches of the pedigree. Several migraine affected individuals in the same family occurred across as many as four generations of the Core Pedigree. Ethical approval has been obtained from the QUT Ethics for Human Research Committee for this study (Approval Number 1400000749).

#### Molecular methods

DNA samples were genotyped using on the Illumina Bead Array 500GX Reader. using Illumina Infinium High Density (HD) Human610‐Quad DNA Bead Chips version 1 as described previously (Cox et al. 2012). A total of 620,901 genome‐wide markers were genotyped and markers had a median spacing of 2.7 kb (mean = 4.7 kb) throughout the genome. In this study all genotyped SNPs were directly observed via lab‐based assays, that is, not imputed. Individuals with a call rate below 95% and SNPs with a call rate below 99%, deviating from Hardy–Weinberg equilibrium (*p*_HWE_<1 × 10^−7^) or with a minor allele frequency of <1% were excluded.

#### Statistical modeling

SNPs located within 10 kb of all known NEMP genes were selected using a custom script developed in R. Gene boundaries were obtained for /Homo sapiens/ (build hg19) as a gff3 file, then filtered using a list of mitochondrial protein names obtained from MitoProteome (Cotter et al. 2004), extending 10 kb in each direction from each gene using a custom perl script. The region of selection was extended by 10 kb to ensure that regulatory regions were included in the selection. The gff3 location file was then intersected with the locations for SNPs genotyped in the NI population using a custom python script that interfaced with HTSeq (Hu et al. 2013; Anders et al. 2015).

In total genotype data for 15351 SNPs representing 956 NEMP genes were selected for 315 key Norfolk Island individuals – 80 migraine sufferers compared to 235 controls (see Table S1 for NEMP gene annotation). A logistic regression model was used to statistically model the variant association with migraine. The model included covariates which were used to adjust for age, sex, and relatedness within the pedigree. Plink v1.07 (Purcell et al. 2007) was used for the logistic regression model. Since this study utilizes a gene‐centric approach and given the relatively small sample size of the discovery cohort we set a relaxed significance threshold of 0.005 for the discovery phase. SNPs which exceeded this suggestive significance threshold were moved forward into the replication phase to assess the validity of findings in an independent, and general population, migraine cohort.

### Replication phase – validation through an independent migraine cohort

#### Sample selection

Migraine patients and controls were recruited from the local South East Queensland region as previously described (Colson et al. 2004). They were all of Caucasian origin, and diagnosed as having MA or MO based on criteria specified by the International Headache Society. An unaffected control group with no family history of migraine was matched for age (± 5 years), sex, and ethnicity. Blood samples obtained from patients were collected through the Genomics Research Centre clinic and DNA was extracted using a salting out method. Approval for the study protocol was acquired from QUT's Ethics Committee (Approval Number 1300000484). In total 1128 individuals comprising 544 patients and 584 controls were genotyped. Migraine sufferers included both MA and MO subtypes, with 381 MA patients and 163 MO patients. As is typical of migraine, samples were skewed in a 3:1 gender ratio with 294 males and 834 females included.

#### Molecular methods

Matrix‐assisted desorption/ionization time‐of‐flight (MALDI‐TOF) mass spectrometry was used to genotype each sample in a multiplexed reaction using the Sequenom (Agena Biosciences) MassARRAY^®^ system (Shchepinov et al. 2001). The time of flight was recorded for each fragment which has a slightly different density according to genotypes, which were visually checked by the cluster plots and called according to strict parameters. Dilution and pooling of annealing and extension primers, amplification and purification steps, desalting of samples, dispensing to chip, and acquiring data through MALDI‐TOF were all carried out according to the manufacturer's instructions.

#### Statistical modeling

Logistic regression modeling was performed in Plink v1.07 (Purcell et al. 2007) to test for association between the 21 SNPs genotyped and migraine susceptibility in a large Australian Caucasian population. The model was adjusted for sex to avoid skewing of results. A regression model was utilized for overall migraine patients as well as the migraine subtypes MA and MO. In total 1128 individuals comprising 544 patients and 584 controls were included in the analysis. Subtype analysis comprised 381 MA patients and 163 MO patients. When correcting for multiple testing, only a single SNP deviated from HWE (rs13361997, *P* = 4.78E‐05). This SNP was disregarded from further analysis. A significance threshold of *P* < 0.005 was also set for the replication cohort.

## Results

### Discovery phase

This study aimed to undertake a gene centric targeted approach in order to specifically identify the role of NEMP genes in relation to migraine susceptibility. In total 15351 SNPs across 956 NEMP genes were selected for testing in 315 Norfolk Island individuals and of these, 67 were found to be suggestively associated with migraine susceptibility (*P* < 0.005). Key genes were identified which contain multiple variants exhibiting association with migraine. These include Sarcosine dehydrogenase (*SARDH),* CUB and Sushi multiple domains 1 *(CSMD1),* Phosphatidylserine decarboxylase *(PISD),* fatty acid elongase 6 *(ELOVL6),* CUB and Sushi multiple domains 3 *(CSMD3)* and casein kinase 1 gamma 3 (*CSNKIG3)*. Genes with three or more nominally associated variants were prioritized for replication as shown in Table 1.

**Table 1:** Discovery of NEMP genetic variants associated with migraine susceptibility in the Norfolk Island population

Chr	Gene	rs ID	Position^a^	Allele	Odds ratio	MAF cases	MAF controls	95% CI lower	95% CI upper	*P* value
8	CSMD1	rs6993396	2924014	T	0.5707	0.2687	0.396	0.3856	0.8152	0.003096
8	CSMD1	rs7828513	3432054	T	0.5825	0.325	0.4414	0.4266	0.87	0.004645
8	CSMD1	rs17066503	3452058	C	3.349	0.06875	0.02087	1.646	7.287	0.001503
8	CSMD1	rs7815959	3460063	C	1.73	0.3438	0.2388	1.164	2.395	0.004444
22	PISD	rs5994415	32004588	A	1.726	0.3812	0.2623	1.218	2.464	0.002542
22	PISD	rs12171042	32011225	C	1.83	0.4375	0.2982	1.298	2.581	0.0006661
22	PISD	rs9956	32015450	C	1.817	0.4375	0.3004	0.6214	1.325	0.0007959
4	ELOVL6	rs11733718	111073867	G	0.5673	0.375	0.5089	0.4098	0.8182	0.001769
4	ELOVL6	rs900328	111074900	C	0.5687	0.375	0.5105	0.4066	0.8139	0.001778
4	ELOVL6	rs7681294	111082310	T	0.5882	0.375	0.5022	0.4209	0.8403	0.003141
8	CSMD3	rs16883344	113282795	A	3.833	0.05	0.01408	1.481	9.163	0.004581
8	CSMD3	rs16883388	113316520	C	6.973	0.03125	0.004735	1.941	23.69	0.00258
8	CSMD3	rs16883751	113500330	A	2.794	0.06875	0.02186	1.578	6.914	0.003875
5	CSNK1G3	rs9327298	122850321	A	1.697	0.35	0.2377	1.205	2.475	0.003648
5	CSNK1G3	rs4530754	122855416	G	0.5767	0.3438	0.473	0.4101	0.8305	0.002708
5	CSNK1G3	rs7705070	122862876	T	1.696	0.35	0.2377	1.203	2.478	0.003838
5	CSNK1G3	rs7737667	122875622	G	1.697	0.35	0.2377	1.205	2.475	0.003648
5	CSNK1G3	rs2052485	122882219	A	0.5863	0.3438	0.4664	0.4216	0.852	0.003828
5	CSNK1G3	rs6595459	122908361	A	0.5863	0.3438	0.4664	0.4216	0.852	0.003828
5	CSNK1G3	rs10037048	122961813	C	0.5848	0.3187	0.4426	0.4115	0.8436	0.003881
9	SARDH	rs2073815	136573412	C	1.683	0.5813	0.4557	1.18	2.33	0.003393
9	SARDH	rs522676	136579589	C	0.485	0.2062	0.3385	0.338	0.7629	0.0007839
9	SARDH	rs916620	136596750	A	0.5372	0.2125	0.3311	0.3642	0.816	0.003012
9	SARDH	rs493901	136600201	C	0.5578	0.35	0.4856	0.4019	0.8096	0.001491

a Genomic position according to build Hg19 of the human genome.

### Replication phase

Leading on from the discovery phase where 67 NEMP SNPs were shown to be suggestively associated with migraine in the Norfolk Island population (*P* < 0.005), variants were chosen for replication to assess the validity of findings in an Australian Caucasian population. In total 1128 individuals comprising 544 patients and 584 controls were genotyped. After analyzing all genotyping results the logistic regression model showed a significant association between several SNPs and migraine susceptibility (*P* < 0.005), replicating the findings in the Norfolk Island population (Table 2). The most significantly associated SNPs were located in the genes *ELOVL6* (*P* = 0.00035), *SARDH* (*P* = 0.00081), and *CSNK1G3* (*P* = 0.00037). After applying a Bonferroni correction, three SNPs located in *ELOVL6, SARDH* and *CSNK1G3* pass the significance threshold suggesting a particularly important role for these genes in migraine susceptibility. The three SNPs which showed significant association were implicated in migraine susceptibility in both the Norfolk Island population and an unrelated migraine patient‐control population, providing clear evidence for a role between the identified variants and migraine susceptibility.

**Table 2:** NEMP genetic variants which show association with migraine in an independent patient‐control population

Chr	SNP	Allele	Odds ratio	MAF cases	MAF controls	*P* value	Gene	Function
4	rs7681294	T	0.6715	0.409	0.4801	0.000349	*ELOVL6*	Intronic variant
9	rs2073815	C	1.434	0.4598	0.3764	0.000808	*SARDH*	Synonymous codon
5	rs9327298	A	0.146	0.005076	0.08613	0.000373	*CSNK1G3*	Intronic variant

## Discussion

Mitochondria function primarily to produce a constant supply of energy to the cells of the body in the form of ATP. The most efficient conversion of calories from our fuel intake (food) into useable energy is through the oxidative phosphorlyative chain under oxidative conditions where glucose is converted to ATP. The main metabolic pathways include glycolysis, the conversion of acetyl‐CoA to GTP and other intermediates through the citric acid cycle, the pentose phosphate pathway, the urea cycle, fatty acid oxidation and gluconeogenesis. Molecules from our dietary intake are metabolized according to their properties and the end products are passed along the OXPHOS units in the mitochondria to produce energy (Wallace 2005).

Reactive Oxygen Species (ROS) are produced as biproducts of the energy conversion process and can have damaging effects on cells if they are allowed to accumulate (McCord 2000). Additional functions of the mitochondria include calcium homeostasis which is critical for neuronal function and initiation of apoptosis. Mitochondria occur in proportion to each tissue's energy requirements with muscle and nervous tissues containing several thousand mitochondria per cell. It has been well established that mitochondrial dysfunction affects the tissues with the highest energy requirements and that the most severe mitochondrial disorders are neuromuscular diseases (Cordeiro et al. 2009). It has been hypothesized that the role of mitochondrial dysfunction in neurological conditions has been overlooked by the medical community and that further scientific investigations in this arena are warranted. This is the first study to comprehensively investigate the role of NEMPs which are critically involved in mitochondrial function in relation to migraine susceptibility (Wallace 2005).

This study identified, for the first time, a link between genetic variants influencing mitochondrial function and migraine susceptibility providing molecular genetic evidence that mitochondrial dysfunction plays an important role in migraine susceptibility. The three key genes identified are important for metabolic pathways and could represent novel therapeutic targets. The discovery phase of this study identified 67 (*P* < 0.005) NEMP variants suggestively associated with migraine susceptibility. Several SNPs were prioritized for replication in an outbred Australian Caucasian migraine population to assess the validity of these findings in a more general context, including multiple variants in the top candidate genes. Genes with three or more nominal associations were included in order to examine the gene as a whole in the migraine disease pathway. The replication study supported significant association with the genes *ELOVL6* (*P* = 0.00035), *SARDH* (*P* = 0.00081), and *CSNK1G3* (*P* = 0.00037) and migraine. In total three SNPs were found to be significant in both the genetically isolated Norfolk Island population and an independent migraine patient‐control cohort. Given the biological plausibility and clear replication in a second population the results support a relationship between NEMP genes and migraine susceptibility.

The most significant finding from the replication study was the association between *ELOVL6* and migraine, a nuclear encoded gene which is transported into the mitochondrial matrix and plays a key role in energy metabolism. Within this key gene, we found rs7681294 to be associated with migraine susceptibility (*P* = 0.000349). The odds ratio of 0.6715 suggests that individuals who carry the C>T change are protected by the T allele. This gene encodes for an enzyme in humans which catalyzes the elongation of saturated and monounsaturated fatty acids with 12, 14 and 16 carbons. It has been found to be expressed in fatty tissues of the body and a recent study has provided evidence for this to be a new candidate gene involved in energy deficiencies with variants in this gene associated with insulin sensitivity, suggesting an important role in metabolic processes (Morcillo et al. 2011).

Significant associations were also found between the NEMP gene *SARDH* (*P* = 0.000808) and migraine susceptibility providing evidence that genetic variation in nuclear encoded genes which are mitochondrially expressed may play an important role in migraine. Sarcosine dehydrogenase situated on chromosome 9 encodes for an enzyme which is localized to the mitochondrial matrix and catalyzes the oxidative demethylation of sarcosine. Mutations in this gene have been associated with sarcosinemia, a mild inborn error of metabolism (Scott et al. 1970). Some reports have suggested severe problems associated with this disease including developmental delay and neurological problems (Meissner and Mayatepek 1997). Casein kinase 1 gamma 3 (*CSNKIG3)* encodes a member of a family of serine/threonine protein kinases that phosphorylate caseins and other acidic proteins. This gene plays an important role in basic human metabolic processes (Davidson et al. 2005).

This is the first molecular genetic study to comprehensively investigate the role of NEMPs in migraine susceptibility and we present empirical evidence for the first time to establish a link between mitochondrial dysfunction and migraine. This study shows a significant link between genes involved in mitochondrial function and migraine in both a genetically isolated population, as well as an outbred Australian Caucasian population. Three new key candidate genes were identified in this study, showing a novel relationship between metabolic pathways and migraine susceptibility.

## Conflict of Interest

The authors declare that they have no conflict of interest.

## Supporting information


**Table S1.** NEMP_Gene_Annotation.
